# Identification and localization of minimal MHC-restricted CD8+ T cell epitopes within the *Plasmodium falciparum *AMA1 protein

**DOI:** 10.1186/1475-2875-9-241

**Published:** 2010-08-24

**Authors:** Martha Sedegah, Yohan Kim, Bjoern Peters, Shannon McGrath, Harini Ganeshan, Jennylynn Lejano, Esteban Abot, Glenna Banania, Maria Belmonte, Renato Sayo, Fouzia Farooq, Denise L Doolan, David Regis, Cindy Tamminga, Ilin Chuang, Joseph T Bruder, C Richter King, Christian F Ockenhouse, Bart Faber, Edmond Remarque, Michael R Hollingdale, Thomas L Richie, Alessandro Sette

**Affiliations:** 1 U.S. Military Malaria Vaccine Program, Naval Medical Research Center, 503 Robert Grant Avenue, Silver Spring, MD 20910-7500, USA; 2 La Jolla Institute for Allergy and Immunology, La Jolla, CA, USA; 3 U.S. Military Malaria Vaccine Program, Walter Reed Army Institute of Research, Silver Spring, MD 20910, USA; 4 Henry M. Jackson Foundation for the Advancement of Military Medicine, Rockville MD 20852, USA; 5 Queensland Institute of Medical Research, Brisbane, Queensland, Australia; 6 GenVec, Gaithersburg, MD 20878, USA; 7 Biomedical Primate Research Centre, Rijswijk, The Netherlands; 8 Consultant to the USMMVP, Malaria Department, NMRC, Silver Spring, MD 20910, USA

## Abstract

**Background:**

*Plasmodium falciparum *apical membrane antigen-1 (AMA1) is a leading malaria vaccine candidate antigen that is expressed by sporozoite, liver and blood stage parasites. Since CD8+ T cell responses have been implicated in protection against pre-erythrocytic stage malaria, this study was designed to identify MHC class I-restricted epitopes within AMA1.

**Methods:**

A recombinant adenovirus serotype 5 vector expressing *P. falciparum *AMA1 was highly immunogenic when administered to healthy, malaria-naive adult volunteers as determined by IFN-γ ELISpot responses to peptide pools containing overlapping 15-mer peptides spanning full-length AMA1. Computerized algorithms (NetMHC software) were used to predict minimal MHC-restricted 8-10-mer epitope sequences within AMA1 15-mer peptides active in ELISpot. A subset of epitopes was synthesized and tested for induction of CD8+ T cell IFN-γ responses by ELISpot depletion and ICS assays. A 3-dimensional model combining Domains I + II of *P. falciparum *AMA1 and Domain III of *P. vivax *AMA1 was used to map these epitopes.

**Results:**

Fourteen 8-10-mer epitopes were predicted to bind to HLA supertypes A01 (3 epitopes), A02 (4 epitopes), B08 (2 epitopes) and B44 (5 epitopes). Nine of the 14 predicted epitopes were recognized in ELISpot or ELISpot and ICS assays by one or more volunteers. Depletion of T cell subsets confirmed that these epitopes were CD8+ T cell-dependent. A mixture of the 14 minimal epitopes was capable of recalling CD8+ T cell IFN-γ responses from PBMC of immunized volunteers. Thirteen of the 14 predicted epitopes were polymorphic and the majority localized to the more conserved front surface of the AMA1 model structure.

**Conclusions:**

This study predicted 14 and confirmed nine MHC class I-restricted CD8+ T cell epitopes on AMA1 recognized in the context of seven HLA alleles. These HLA alleles belong to four HLA supertypes that have a phenotypic frequency between 23% - 100% in different human populations.

## Background

The sterile protective immunity to malaria induced in humans by immunization with irradiated sporozoites is thought to be mediated by CD4+ and CD8+ T cells responding to malaria peptides expressed on the surface of hepatocytes or antigen presenting cells by secreting interferon-gamma (IFN-γ) and/or by cytotoxic responses, although anti-sporozoite antibodies may contribute [1-5]. Many sporozoite and liver stages antigens have been identified [6] that could play a role in sporozoite and liver stage immunity, including the circumsporozoite protein (CSP), the main antigenic component of the partially protective RTS, S vaccine currently undergoing Phase 3 testing in sub-Saharan Africa[7,8]. Although CSP contributes to the protection induced by irradiated sporozoites, it is not required, indicating the importance of other antigens[9,10]; additionally it has not been possible to consistently induce CD8+ T cell responses using recombinant CSP-based vaccines such as RTS, S[7,11,12]. Combining CSP with other pre-erythrocytic stage antigens and using a vaccine platform such as adenovirus vectors better able to induce class I restricted cell-mediated immunity might therefore more effectively target the hepatic stages of infection and reproduce the immunity induced by the irradiate sporozoite vaccine.

Apical Membrane Antigen-1 (AMA1) is a candidate antigen for inclusion with CSP in a multi-antigen malaria vaccine. AMA1 has previously been tested in several clinical trials as a recombinant protein and elicited both CD4+ and CD8+ T cell responses[13-18]. AMA1 is an integral membrane protein found in all species of *Plasmodium *and has traditionally been regarded as a blood stage antigen, since it is required for the invasion of red blood cells[19], monoclonal and polyclonal antibodies targeting AMA1 inhibit blood stage growth *in vitro*, naturally acquired anti-AMA1 antibodies correlate with protection against clinical malaria in endemic areas [20-24], and vaccines based on AMA1 elicit protection against blood stage infection [13,25] in animal models that appears to be antibody mediated [25,26]. However, AMA1 is also expressed in sporozoites and liver stage parasites[27], and thus may be a suitable target for CD8+ T cell responses directed toward liver stage parasites.

To test this hypothesis, two adenovirus-vectored vaccines encoding *P. falciparum *CSP and AMA1 were evaluated in a Phase 1 clinical trial. Volunteers were administered a single dose of the mixed CSP- and AMA1-encoding constructs (termed the NMRC-M3V-Ad-PfCA vaccine), either 2 × 10^10 ^(1 × 10^10 ^of each construct) or 1 × 10^11 ^(5 × 10^10 ^of each construct) particle units (pu). Robust CD4+ and CD8+ T cell responses were induced in both low dose and high dose groups against both antigens, as measured by *ex vivo *enzyme-linked immunospot (ELISpot) assay conducted using pools of 15-mer peptides spanning full length CSP or AMA1 as the stimulant. These responses were significantly higher in the low dose than the high dose group, and the vaccine consistently induced stronger CD8+ than CD4+ T cell responses in both groups (Sedegah M, Tamminga C, McGrath S, House B, Ganeshan H, Lejano J, Abot E, Banania GJ, Sayo R, Farooq F, Belmonte M, Manohar N, Richie NO, Wood C, Long CA, Regis D, Shi M, Chuang I, Spring M, Epstein JE, Mendoza-Silveiras J, Limbach K, Patterson NB, Bruder JT, Doolan DL, King CR, Soisson L, Diggs C, Carucci D, Dutta S, Hollingdale MR, Ockenhouse CF, Richie TL. Multi-stage adenovirus 5-vectored falciparum malaria vaccine elicits CD8+ and CD4+ T cell responses and limited antibodies in healthy, seronegative adults, submitted). The CD8+ T cell IFN-γ responses induced in this clinical trial provided the opportunity to identify the underlying minimal CD8+ T cell epitopes. Frozen peripheral blood mononuclear cells (PBMC) collected from five volunteers from the more immunogenic low dose group were therefore selected for this epitope mapping study.

The crystal structure of the AMA1 ectodomain shows a conserved central core and variable external loops formed by Domains I, II and III[28]. About 10% of amino acids are polymorphic and many of these cluster within the tertiary structure[28-32] on one external surface and are presumably accessible to antibodies[31,32]. The most polymorphic regions surround a hydrophobic groove containing cryptic and conserved epitopes[31,32]. A recent study in Mali identified 186 unique AMA1 haplotypes largely varying at these polymorphic sites[33], while other studies have shown minimal cross-reactivity among the various allelic variants[17]. This immune diversity would appear to represent a major barrier to developing antibody-based vaccines against AMA1 [26,31,34]. The inhibitory MAb 1F9 maps to variable epitopes[24] including one site that is also recognized by a putative receptor for AMA1 binding to red blood cells[35]. Less is known regarding the most important T cell epitopes of AMA1, and whether or not they fall in the regions of highest variability. Previously identified proliferative AMA1 epitopes in malaria-exposed individuals in Kenya were mapped to both variable and non-variable regions [36].

Approaches to mapping B and T epitopes have included using antibodies[37-42], peptides, cell arrayed polypeptides or phages[36,43-48], computer-based algorithms [49-53] such as NetMHC used in this study[54], and combinations of these approaches[55]. In this study, peptide-based mapping and NetMHC algorithms were combined to identify class I-restricted AMA1 epitopes. Peptide pools showing positive responses in ELISpot assays using PBMC from the five most responsive volunteers from the low dose group were deconvoluted by testing each individual 15-mer peptide. Next, computerized algorithms in NetMHC software[56] were used to predict the binding affinities of AMA1 8-10-mer sequences within the dominant peptides for defined HLA-A or HLA-B supertypes expressed by each immunized volunteer. A subset of the predicted epitopes was synthesized, and ELISpot depletion and intracellular cytokine staining (ICS) assays were performed to confirm recall responses by PBMC from the immunized volunteers and to show their CD8+ T cell-dependence. A pool of selected minimal epitopes provided a potentially suitable reagent to efficiently measure anti-AMA1 CD8+ T cell responses in genetically diverse populations. Finally, 10 of the 14 epitopes were localized to the tertiary structure of AMA1.

## Methods

### Ethics

This study was conducted according to the Declaration of Helsinki and the U.S. Code of Federal Regulations regarding the protection of human participants in research including The Nuremberg Code, The Belmont Report, 32 CFR 219 (The Common Rule) and all regulations pertinent to the Department of Defense, the Department of the Navy, the Department of the Army, the Bureau of Medicine and Surgery of the United States Navy and the internal policies for human subject protections and the standards for the responsible conduct of research of the Naval Medical Research Center (NMRC) and US Army Medical Research and Materiel Command (USAMRMC). NMRC holds a Department of Defense/Department of the Navy Federal Wide Assurance for human subject protections, and a Federal Wide Assurance (FWA 00000152) from the Office for Human Research Protections (OHRP) for cooperation with the Department of Health and Human Services. All NMRC key personnel are certified as having completed mandatory human research ethics education curricula and training under the direction of the NMRC Office of Research Administration (ORA) and Human Subjects Protections Program (HSPP). The trial was performed under US Food and Drug Investigational New Drug Application BB-IND-13003.

### Vaccine and trial design

The NMRC-M3V-Ad-PfCA vaccine used in this study is a combination of two separate recombinant adenovirus 5 constructs, one expressing full length *P. falciparum *CSP (minus 16 repeats, and insertion of 23 amino acids derived from the 3'-noncoding bovine growth hormone polyadenylation sequence at the C-terminus) and the other expressing full length *P. falciparum *AMA1 (both strain 3D7). Epitope mapping was conducted using PBMC taken from volunteers 1 month after administration of one intramuscular injection of 2 × 10^10 ^particle units (pu) of the combination vaccine, unless other intervals are indicated.

### Volunteers and HLA typing

Five of the six immunized volunteers in the low dose group (v001, v002, v005, v008 and v012) were used in this study, as v006 gave poor post-immunization responses. Low to moderate resolution HLA molecular typing for HLA-A and HLA-B loci (Department of Defense Bone Marrow Donor Program using specific oligonucleotide probes to amplify HLA class I and II genes) provided a list of allelic codes from which it was possible to tentatively assign each volunteer to an HLA-A or HLA-B supertype using code lists http://bioinformatics.nmdp.org/HLA/Allele_Codes/Allele_Code_Lists/index.html (Table 1).

**Table 1:** Volunteer HLA A and B supertypes

Volunteer	A Supertype	B Supertype
001	A01/A02	B44/B44
002	A01/A02	B08/B44
005	A01/A02	B08/B27
008	A02/A03	B27/B27
012	A01/A03	B44/B58

Low resolution molecular HLA typing permitted identification of the HLA supertype for each volunteer.

### PBMC

PBMC cryopreserved in liquid nitrogen induced comparable ELISpot activity as fresh cells (data not shown) and were used in these experiments. Due to limitation of the samples collected at 1 month post immunization, PBMC collected at other time points were used in some cases (as indicated), as long as responses were still moderately strong.

### Peptides and peptide pools

153 15-mer peptides overlapping by 11 amino acids and spanning the length of AMA1 were synthesized commercially (Mimotopes, VIC, Australia, >80% purity) and grouped into 12 peptide pools containing 10 to 13 peptides each (Table 2). Seven of these pools (Ap1, Ap3, Ap4, Ap7, Ap8, Ap10 and Ap11) containing 91 peptides elicited strong ELISpot responses among the volunteers. Proliferative T cell epitopes previously identified in Kenya[36,43] are shown for each peptide pool. All 91 peptides were assayed individually, identifying 16 individual 15-mers showing positive ELISpot responses. Fourteen predicted 8-10-mer epitopes identified within these 16 15-mers were synthesized (Alpha Diagnostics Intl. Inc., San Antonio, TX, USA >91% purity) and tested for recall responses. The positive control was commercially obtained Class I Peptide Pool *Plus *(Anaspec, USA). Negative control was media with all supplements but no antigen-specific stimulant.

**Table 2:** AMA1 peptides used in ELISpot and ICS assays

Pool	Amino acids	Number peptides	Class II T epitope	Residues	T epitope sequence
**Ap1**	1-63	13	PL186	14-35	EFTYMIFNGRGQNYWEHPYQKS
			PL187	41-51	INEHRPKEY
Ap2	53-115	13	PL188	92-103	NLFSSIEIVERS
**Ap3**	105-167	13			
**Ap4**	157-219	13	PL189	188-204	PLMSPMTLDEMRHFYKD
Ap5	209-271	13	PL190	218-229	SRHAGNMIPDND
			PL191	259-271	NGPRYCNKDE
Ap6	261-323	13	PL192	279-288	AKDISFQNYT
**Ap7**	313-375	13	PL171	348-366	DQPKQYEQHLTDYEKIKEG
**Ap8**	365-427	13	PL193	390-402	YKSHGKGYNWGNY
Ap9	417-479	13	PL172	444-461	SLYKNEIMKEIERESKRI
**Ap10**	469-531	13			
**Ap11**	521-583	13	PL194	527-538	EYKDEYADIPEH
			PL173	571-588	GNAEKYDKMDEPQHYGKS
Ap12	573-622	10	PL173	571-588	GNAEKYDKMDEPQHYGKS

PfAMA1 peptide sequences and residue numbers were based on those of the *P. falciparum *clone FC27 Gene Bank ID 810891. Previously identified class II AMA1 epitopes (see text) were distributed among peptide pools, except pools 3 and 10. Seven immunodominant pools are indicated in bold type.

### Ex vivo IFN-γ ELISpot assays

IFN-γ ELISpot assays were conducted as previously described[57]. Cryopreserved PBMC were suspended in 100 μL complete medium and stimulated with AMA1 peptides in 100 μL of complete medium at a final concentration of 10 μg/mL of each peptide tested[57]. Cultures were incubated for 36 hours at 37°C, 5% CO2. Depending on availability of cells, each PBMC sample was assayed in duplicate, triplicate, or quadruplicate and the number of IFN-γ-secreting spot forming cells (sfc) was counted using an automated ELISpot reader (AID, GmbH, Germany). In duplicate assays, all values were used in analysis. For triplicate or quadruplicate assays, outliers were rejected if any single value contributed more than 50% of the standard deviation of the replicates and if its value was three-fold greater or three-fold less than the average of the remaining two (or three) values. The mean number of sfc obtained in negative control wells was subtracted from the value of each test well from the same sample. Negative counts generated by this background subtraction were converted to zero. The mean number of spots of the test sample was then calculated and expressed as sfc/million (sfc/m). Based on testing five volunteers with 91 15-mer peptides, 40 sfc/m was used as a conservative cut-off for determining positive activity.

### Characterization of ELISpot IFN-γ-producing cells by T cell subset depletions

PBMC were depleted of T cell subsets using anti-human CD4+ or anti-CD8+ coated Dynabeads M-450 (Dynal, Great Neck, NY) following the manufacturer's instructions. Mock depletion was done with Dynabeads coated with sheep anti-mouse IgG. Flow cytometry confirmed that T cell subset depletions were >99% in all experiments. Data are presented as the sfc/m and percent decrease or increase in activity after depletion.

### Intracellular cytokine staining (ICS)

ICS was performed as published previously [58]. Cryopreserved PBMC were thawed, washed, and resuspended at 1 × 10^7 ^cells per mL in complete medium. Peptides were used at 10 μg/mL and costimulatory antibodies anti-CD28 and anti-CD4+9 d (BD Bioscience, San Jose, CA) were used at 1 μg/mL. Stimulants were added to cells and incubated at 37°C with 5% CO2 for 2 hours. Cells were stained with anti-CD3, anti-CD4+, anti-CD8+, anti-IFN-γ, anti-TNF-α, and anti-IL2 and the entire available sample was acquired on a BD LSRII using FACSDiVa (BD Bioscience) software. Data were analysed using FlowJo Software (Treestar, Inc.). The gating strategy involved progressively measuring total cells; viable cells; lymphocytes; T cells; CD4+ CD8+ populations; and finally a specific cell type expressing a specific cytokine. Results were transferred to Prism (GraphPad) for graphing and statistical analysis. Data for peptides were corrected for media responses. Results are expressed as total IFN- γ from all VD4+ or CD8+ T cells containing IFN- γ either alone or with other cytokines.

### NetMHC-based epitope predictions and epitope down-selection

NetMHC[59] was used to predict HLA class I binding affinities of 15-mer peptides. NetMHC returns predicted binding affinity scores that approximate the half maximal inhibitory concentration (IC_50_) in nM. Thus, smaller IC_50 _values indicate stronger binding. Peptides with measured binding affinities less than 500 nM IC_50 _are considered binders.

Because lengths of typical peptides that bind to HLA class I molecules range from 8 to 10-mers, binding predictions were made for all possible 8-10-mers in a 15-mer. In order to discover a minimal epitope for each 15-mer that induced a CD8+ response, the peptide with the strongest predicted binding affinity for a given HLA allele was selected.

In practice, because of the low resolution of HLA typing data available for the volunteers, peptide binding predictions were made for all alleles that matched the provided data. For example, the HLA typing data indicates that volunteer v001 has an allele that belongs in the A02 supertype. The HLA typing data also provided a list of possible alleles that belong in the same supertype, which includes A*0201, A*0207 and A*0209. Peptide binding predictions were made for each allele, provided a predictor was available. Thus, for each 15-mer, the strongest predicted binder out of all 8-10-mer peptides across different possible alleles and its predicted binding affinity were recorded. This strongest predicted binder is referred to as the predicted minimal epitope. In the end, a single predicted minimal epitope was associated with each 15-mer after ranking candidates by binding affinity predictions.

### Epitope mapping on the AMA1 3 D structure

The *P. falciparum *AMA1 Domains I and II model 1Z40 was fitted onto the *P. vivax *AMA1 Domain I, II and III model 1w81 (E chain) using Swiss Pdb-Viewer software [Swiss Institute of Bioinformatics (Basel, Switzerland)]. The final 3 D model was generated by combining Domains I and II of the *P. falciparum *AMA1 model with Domain III of the *P. vivax *AMA1 model, omitting Domains I and II of the *P. vivax *AMA1 model. Accessibility of the amino acid residues was determined by the same software.

## Results

### Volunteers

The five volunteers used in this study expressed a total of seven supertypes (Table 1), A01, A02, A03, B08, B27, B44 and B58, representing three of the six HLA-A and four of the six HLA-B supertypes[60]. Together these cover 100% of the Caucasian population, 27% or the African American population, and a variable per cent of sub-Saharan populations according to their genetic diversity[61]. There were no volunteers with other high frequency supertypes such as A24 or B07[61].

### Identification of AMA1 15-mer peptides most active in ELISpot assay

The 91 15-mers contained within peptide pools Ap1, Ap3, Ap4, Ap7, Ap8, Ap10 and Ap11 were tested individually by ELISpot assay using PBMC from 1 month post immunization. Sixteen peptides were recognized by one or more of the five volunteers at levels > 40 sfc/m, and assigned numbers of 1-16 (Table 3). Some were recognized by more than one volunteer such as peptide 14 in pool 8 (Ap8-11) that was recognized by v001, v005 and v008.

**Table 3:** ELISpot IFN-γ activity of AMA1 peptide pools and individual 15-mer peptides within these pools

Pool	**Vol**.	sfc/m	15-mer peptide	Amino acid number	Sequence	sfc/m	Peptide number
Ap1	001	119	Ap1-3	9-23	LLSAFEFTYMINFGR	48	1
Ap1	001	119	Ap1-9	33-47	QNSDVYRPINEHREH	48	2
Ap1	002	161	Ap1-11	41-55	INEHREHPKEYEYPL	180	3
Ap1	005	325	Ap1-11		INEHREHPKEYEYPL	322	
Ap1	002	161	Ap1-12	45-59	REHPKEYEYPLHQEH	66	4
Ap1	005	325	Ap1-12		REHPKEYEYPLHQEH	118	
Ap1	001	119	Ap1-13	49-64	KEYEYPLHQEHTYQQ	42	5
Ap4	005	208	Ap4-5	173-187	NQYLKDGGFAFPTE	50	6
Ap4	002	208	Ap4-9	189-203	LMSPMTLDEMRHFYK	189	7
Ap4	005	310	Ap4-9		LMSPMTLDEMRHFYK	395	
Ap4	002	208	Ap4-10	193-207	MTLDEMRHFYKDNKY	82	8
Ap4	005	208	Ap4-10		MTLDEMRHFYKDNKY	118	
Ap4	005	208	Ap4-11	197-211	EMRHFYKDNKYVKNL	65	9
Ap7	001	131	Ap7-3	310-324	EDIPHVNEFPAIDLF	139	10
Ap7	001	131	Ap7-4	314-328	HVNEFPAIDLFECNK	96	11
Ap7	001	131	Ap7-7	336-350	CNKLVFELSADQPK	77	12
Ap8	012	138	Ap8-6	384-398	FKADRYKSHGKGYNW	143	13
Ap8	001	78	Ap8-11	405-419	ETQKCEIFNVKPCL	48	14
Ap8	005	156	Ap8-11		ETQKCEIFNVKPCL	218	
Ap8	008	78	Ap8-11		ETQKCEIFNVKPCL	48	
Ap8	005	156	Ap8-12	409-423	CEIFNVKPTCLINNS	127	15
Ap10	001	172	Ap10-13	517-531	TSNNEVVVKEEYDE	233	16
Ap10	012	193	Ap10-13		TSNNEVVVKEEYDE	250	

A total of 16 individual peptides were identified from a total of 91 15-mer peptides from seven active pools. Peptides Ap1-11, Ap1-12, Ap4-9, Ap4-10, Ap8-11 and Ap10-13 stimulated strong responses in more than one volunteer.

### ELISpot assays with 15-mer peptides after depletion of CD4+ and CD8+ T cells

In these depletion studies, the availability of PBMC from 1 month following immunization limited assays to five of the sixteen 15-mers and four of the volunteers listed in Table 3 (v008 excluded). CD8+ T cell depletion reduced ELISpot activity by 56-100% (Table 4) demonstrating the presence of at least one minimal CD8+-restricted epitope within each 15-mer tested for each of the four volunteers that was recognized in the context of supertypes A1, B44 or B08. As shown in Table 3, peptide 3 was recognized by v002 and v005, and peptide 16 by v001 and v012, and this is attributed to common HLA alleles between these volunteers (Table 1). In those cases where depletion of CD4+ T cells also reduced ELISpot responses, the effect was much smaller (0-57%) than for CD8+ T cells, suggesting that these peptides contain predominantly CD8+ T cell epitopes. Depletion of CD4+ T cells led to an increase in ELISpot activity with v001, suggesting the removal of suppressor mechanisms.

**Table 4:** ELISpot IFN-γ activity of AMA1 15-mer peptides after depletion of CD4+ and CD8+ T cells

**Vol**.	Pool	Peptide number*	Sequence	Control depletion sfc/m	CD4 depletion sfc/m (%)	CD8 depletion sfc/m (%)
001	Ap1			155	222 (+43%)	55 (-65%)
		1	LLSAFEFTYMINFGR	85	58 (-32%)	37 (-56%)
		5	KEYEYPLHQEHTYQQ	72	168 (+133%)	17 (-76%)
		16	TSNNEVVVKEEYDE	103	252 (+148%)	13 (-88%)
002	Ap1			187	148 (-21%)	5 (-97%)
		3	INEHREHPKEYEYPL	185	152 (-18%)	11 (-94%)
005	Ap1			267	173 (-35%)	11 (-96%)
		3	INEHREHPKEYEYPL	233	145 (-37%)	0 (-100%)
	Ap4			273	117 (-57%)	0 (-100%)
		7	LMSPMTLDEMRHFYK	204	137 (-32%)	1 (-100%)
012	Ap10			48	37 (-23%)	0 (-100%)
		16	TSNNEVVVKEEYDE	79	90 (+14%)	0 (-100%)

The percent change in ELISpot activity is shown after depletion of CD4+ or CD8+ T cells. Depletion of CD8+ T cells reduced ELISpot activity by 56-100%, whereas CD4+ T cell depletion reduced activity by no more than 57%. Although this suggested that the primary epitopes within these peptide pools and 15-mers are CD8+ T cell epitopes, some peptides may also contain CD4+ T cell epitopes. v001 and v012 showed an increase in ELISpot activity after CD4+ T cell depletion that may indicate the removal of regulatory T cells that had been suppressing CD8+ T cell activity.

* See last column of Table 3.

### NetMHC prediction of class I-restricted epitopes in AMA1 15-mer peptides

NetMHC predicted a series of HLA-A- and B-restricted 8-10-mer epitopes within the 16 15-mers active in ELISpot assays (Table 5). Each predicted epitope was restricted by a specific HLA allele but since the HLA typing of the volunteers was low to moderate resolution, this study selected the best binders for the several candidate alleles within each volunteer's HLA supertype. For example, peptide 1 contained a predicted epitope restricted by HLA B*1801 that matched the v001 HLA supertype B44 as shown in Table 5. Since overlapping peptides were used, in three cases predicted epitopes were contained in more than one peptide, for example epitope E3 was contained in two overlapping peptides 3 and 4 (Table 5). Furthermore, there was also one case where one 15-mer peptide, peptide 14, contained 2 predicted epitopes, E11 and E12, leaving a net of 14 unique epitopes.

**Table 5:** Predicted CD8+ T cell-restricted epitopes within AMA1 15-mer peptides specific for each volunteer

Peptide number	Predicted epitope	Amino acid number	IC_50 _nM	HLA restriction	HLA supertype	Epitope number
1	LLSA**FEFTYMINF**GR	13-21	5	B*1801	B44	E1
2	QN**SDVYRPINEH**REH	35-44	14991	B*4402	B44	E2
3	INEHRE**HPKEYEYPL**	47-55	18	B*0801	B08	E3
4	RE**HPKEYEYPL**HQEH					
5	KE**YEYPLHQEH**TYQQ	51-59	39	B*1801	B44	E4
6	NQ**YLKDGGFAF**PTE	175-183	142	B*0801	B08	E5
7	LMSPM**TLDEMRHFYK**	194-202	17	A*0101	A01	E6
8	M**TLDEMRHFYK**DNKY					
9	E**MRHFYKDNKY**VKNL	198-207	4742	A*0101	A01	E7
10	EDIPHV**NEFPAIDLF**	327-335	7	B*1801	B44	E8
11	HV**NEFPAIDLF**ECNK					
12	CN**KLVFELSA**DQPK	339-346	5708	A*0201	A02	E9
13	FKAD**RYKSHGKGY**NW	389-397	201	A*3002	A01	E10
14	**ETQKCEIFNV**KPCL	405-414	142	A*6802	A02	E11
	E**TQKCEIFNV**KPCL	406-414	919	A*0201	A02	E12
15	C**EIFNVKPTCL**INNS	410-419	617	A*6802	A02	E13
16	TSN**NEVVVKEEY**DE	520-528	15	B*1801	B44	E14

The 15-mer peptides that were recognized by the volunteers (Table 3) were analysed by NetMHC web-based software to predict potential high affinity HLA binding by minimal CD8+ T cell epitopes within each 15-mer. Each minimal epitope was specific for a known HLA allele within each supertype. Those minimal epitopes with the strongest binding affinities for the HLA supertype of each volunteer were selected. The Table shows the minimal epitopes in bold. Two different HLA-restricted epitopes were predicted with peptide 14.

Nine of the 14 resulting epitopes (64%) were classified as strong binders (<500 nM). Of these 14 minimal epitopes, two were predicted to be restricted by HLA-A*0101 and one by HLA-A*3002 (A01 supertype); two by HLA-A*0201 and two by HLA-A*6802 (A02 supertype); two by HLA-B*0801 (B08 supertype); four by HLA-B*1801 and one by HLA-B*4402 (B44 supertype). Epitopes predicted to bind to supertypes A03, B27 and B58 present in the volunteers were not identified.

### ELISpot activity of predicted minimal epitopes compared to parent peptide pool

PBMC available from bleeds at 4 months had similar activity to those at 1 month for v001 and v002 and were, therefore, used for the evaluation of epitopes E1, E2, E3, E4, E6, E8, E9, E12 and E14. ELISpot activity of these predicted minimal epitopes compared favorably to the ELISpot activity of parent peptide pool activity for all nine (Table 6). Availability of PBMC from v005 and v008 was more restricted, and their ELISpot activity was too low to allow evaluation of E5, E7, E11 and E13. PBMC from v012 taken 7 months post-immunization confirmed positive responses for E8 and E14 but suggested that E10, where responses were ten-fold less than the parent peptide pool and below the cut-off of 40 sfc/m, did not constitute the minimal epitope for that volunteer. Therefore, nine of the predicted epitopes were recognized by one or more volunteers, four could not be adequately tested and one was not recognized. Of the nine confirmed epitopes, six had strong predicted binding affinities (< 500 nM) and three were >500 nm (Table 5).

**Table 6:** ELISpot IFN-γ activity of the original peptide pool and the derived 8-10-mer epitopes

Epitope number	Sequence	HLA supertype	sfc/m	Pool	sfc/m	**Vol**.
**E1**	**FEFTYMINF**	B44	121^4m^	Ap1	133^4m^	001
**E2**	**SDVYRPINEH**	B44	114^4m^	Ap1	133^4m^	001
**E3**	**HPKEYEYPL**	B08	69^4m^	Ap1	96^4m^	002
**E4**	**YEYPLHQEH**	B44	222^4m^	Ap1	133^4m^	001
E5	YLKDGGFAF	B08	9^10d^	Ap4	46^10d^	005
**E6**	**TLDEMRHFYK**	A01	51^4m^	Ap4	114^4m^	002
E7	MRHFYKDNKY	A01	15^10d^	Ap4	46^10d^	005
**E8**	**NEFPAIDLF**	B44	224^4m^	Ap7	200^4m^	001
-	-	-	49^7m^	Ap7	80^7m^	012
**E9**	**KLVFELSA**	A02	169^4m^	Ap7	200^4m^	001
E10	RYKSHGKGY	A01	11^7m^	Ap8	108^7m^	012
E11	ETQKCEIFNV	A02	21^10m^	Ap8	57^10m^	008
**E12**	**TQKCEIFNV**	A02	172^4m^	Ap8	104^4m^	001
E13	EIFNVKPTCL	A02	37^10m^	Ap8	57^10m^	008
**E14**	**NEVVVKEEY**	B44	344^4m^	Ap10	155^4m^	001
-	-	-	104^7m^	Ap10	151^7m^	012

Synthetic peptide 8-10-mers were tested in ELISpot assay using PBMC collected 10 days (v005), 4 months (v001, v002), 7 months (v012) or 10 months (v008) following immunization (PBMC collected at 1 month were no longer available). Activity was measured as sfc/m. Nine of the 14 epitopes (E1, E2, E3, E4, E6, E8, E9, E12 and E14) were confirmed as positive using the parent peptide pool as controls, in most cases recalling responses equivalent to those recalled by the parent peptide pool (epitope number and sequences in bold). Four of the epitopes (E5, E7, E11, E13) did not meet the 40 sfc/m cut-off but these results were not considered meaningful as the responses of the parent peptide pools were only marginally positive. The parent peptide pool for E10 recalled 108 sfc/m while the responses to the peptide were much lower (11 sfc/m), suggesting that E10 may not be correctly identified. Assays where the parent peptide pool did not exceed the cut-off of 40 sfc/m were not included in the table (see Methods).

### ICS CD4+ and CD8+ T cell total IFN-γ responses to AMA1 peptide pools and epitopes

**Figure 1:**
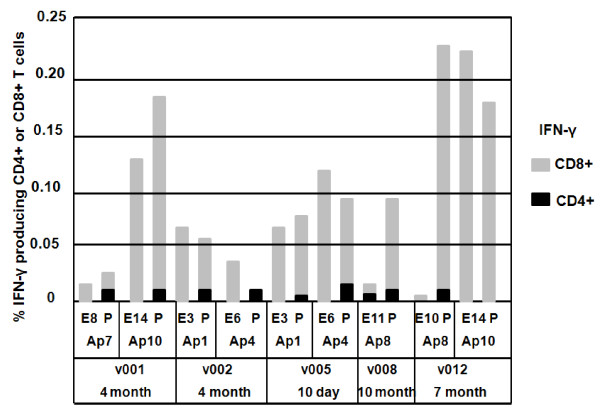
**AMA1 peptide pools and minimal epitopes elicit ICS CD4+ and CD8+ T cell IFN-γ responses**. AMA1 peptide pools (P) and associated minimal CD8+ T epitopes (E) were tested in ICS assays using PBMC collected 10 days (v005), 4 months (v001, v002), 7 months (v012) or 10 months (v008) following immunization. Activity was measured as % cytokine producing CD4+ and CD8+ T cells producing IFN-γ.

PBMC from the same time points were used in ICS assays to measure total IFN-γ from CD4+ and CD8+ T cell subsets in response to selected minimal epitopes and parent peptide pools (Figure 1). These assays showed that the frequency of CD8+ T cells producing total IFN-γ was far greater than that of CD4+ T cells. Four epitopes (E3, E6, E8 and E14) induced equal or more CD8+ IFN-γ than the parent peptide pool, whereas two epitopes (E10 and E11) induced weak CD8+ IFN-γ responses. PBMC were insufficient to perform assays for the other epitopes. These results supported the identification, as minimal MHC class I epitopes, of E3, E6, E8 and E14, while E10 and E11 could not be confirmed.

### ICS CD4+ and CD8+ T cell multifunctional responses to AMA1 peptide pools and minimal epitopes

**Figure 2:**
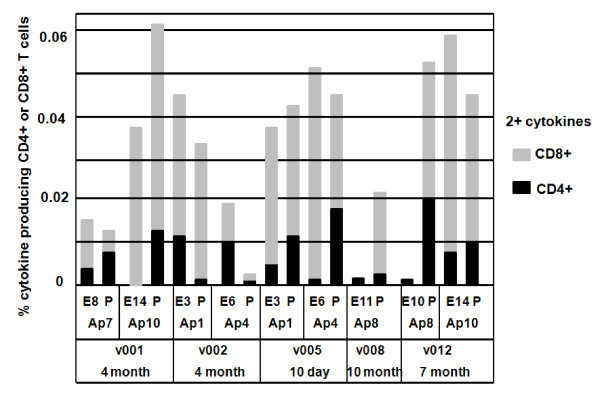
**AMA1 peptide pools and minimal epitopes elicit ICS CD4+ and CD8+ T cell multifunctional responses**. AMA1 peptide pools (P) and associated minimal CD8+ T epitopes (E) were tested in ICS assays using PBMC collected 10 days (v005), 4 months (v001, v002), 7 months (v012) and 10 months (v008) following immunization. Multifunctional activity was measured as % cytokine producing CD4+ and CD8+ T cells producing at least two cytokines among IFN-γ, TNF-α and IL-2.

The pattern of multifunctional CD8+ T cell responses (defined as cells expressing two or more cytokines) recalled by each minimal epitope (Figure 2) were generally similar to that of total CD8+ IFN-γ responses (Figure 1), although they were lower. Concordant with total IFN-γ responses, epitopes E10 and E11 did not appear to induce multifunctional responses with v008 and v012.

### ELISpot assays with epitope mixture AMA1-14e, single AMA1 pool, individual pools and individual epitopes

**Figure 3:**
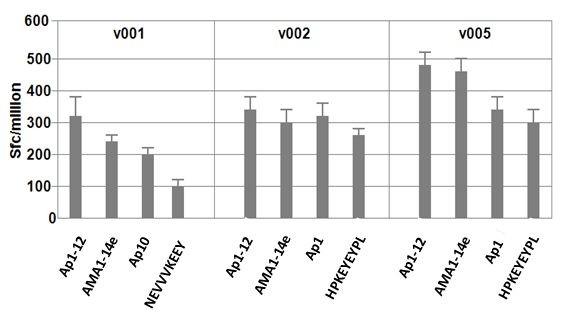
**AMA1-14e is as active in ELISpot as peptide pools and minimal epitopes**. PBMC from each volunteer were tested in ELISpot assays with Ap1-12 (mixture of 153 15-mer overlapping peptides), AMA1-14e (mixture of 14 minimal epitopes), Ap1 or Ap10, and individual epitopes (consistent with that volunteer's HLA supertype - NEVVVKEEY is E14 and HPKEYEYPL is E3). AMA1-14e was as active as Ap1-12 with v002 and v005 and nearly as active with v001. Bars represent standard deviation of the mean response (duplicates).

The feasibility of using pooled epitopes to evaluate CD8+ T cell responses to AMA1 was investigated to establish that non-binding epitopes did not block specific HLA-restricted binding. Three volunteers who consistently gave high responses to peptide pools were tested with four different stimulants: (1) Ap1-Ap12: all 153 15-mer peptides at 1.25 μg/mL; (2) AMA1-14e: the 14 predicted epitopes mixed and tested at 10 μg/mL; (3) 15-mer peptide pools at 10 μg/ml that were strongly recognized by these volunteers; (4) The predicted epitope at 10 μg/ml within those selected 15-mer peptide pools previously shown to have ELISpot activity. As shown in Figure 3, AMA1-14e was as active or nearly as active in inducing ELISpot responses as a mixture of all 153 15-mers (Ap1-Ap12).

### Phenotype of cells involved in ELISpot and ICS assays with epitope mixture AMA1-14e

**Figure 4:**
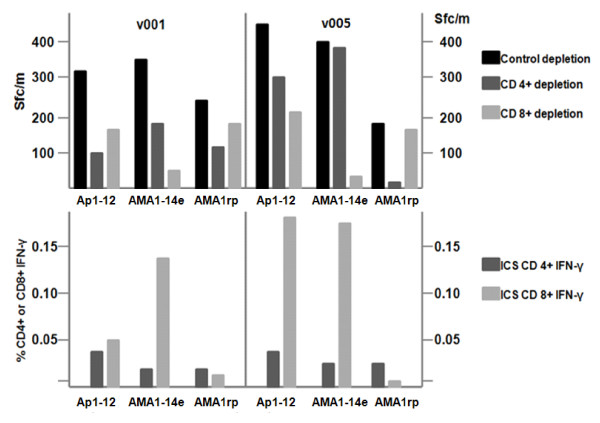
**ELISpot activity against AMA1-14e after CD4+ or CD8+ T cell depletion**. PBMC from v001 and v005 were tested in IFN-γ ELISpot assays after CD4+ or CD8+ T cell depletion (top panel), and in ICS assay for total % CD4+ or CD8+ T cell IFN-γ (lower panel), with Ap1-12 (all 153 15-mer peptides), AMA1-14e, and AMA1 recombinant protein (AMA rp). ELISpot depletion and ICS assays show that AMA1-14e and Ap1-12 are both strongly recognized by CD8+ T cells. However, recombinant AMA1 protein is preferentially recognized by CD4+ T cells.

CD8+ T cell depletion reduced ELISpot activity to AMA1-14e by about 85% - 95% in the two volunteers tested, v001 and v005, and AMA1-14e much more strongly induced CD8+ than CD4+ T cell IFN-γ by ICS assay. In contrast, Ap1-Ap12 induced both CD4+ and CD8+ T cell responses, whereas AMA1 recombinant protein only recalled CD4+ T cell responses (Figure 4). Thus AMA1-14e may be a suitable reagent to demonstrate CD8+ T cell immunogenicity of AMA1-based vaccines in vaccine trials where volunteers have HLA alleles matching the epitopes in AMA1-14e.

### Localization of the predicted minimal epitopes within AMA1

E1 lies within the signal domain, E2, E3, E4 within the prodomain, E5, E6 and E7 within Domain I, E8, E9, E10, E11, E12 and E13 within Domain II, and E14 within Domain III (Table 7). Three of the 14 predicted CD8+ minimal epitopes were fully or partially within proliferative epitopes identified in Kenya: E1 and PL186; E6 and PL189; E10 and PL193. The other 11 predicted epitopes were largely or wholly independent of these proliferative epitopes.

**Table 7:** Summary of the properties of AMA1 epitopes

Epitope	HLA-restriction	Amino acids	Sequence	Location	Crystal face	Accessibility
**E1**	B44	13-21	FEFTYMINF	Signal		
**E2**	B44	35-44	S**D**VY**R**P**I**NEH	Prodomain		
**E3**	B08	47-55	H**PKE**Y**E**Y**P**L	Prodomain		
**E4**	B44	51-59	Y**E**Y**P**LHQ**E**H	Prodomain		
E5	B08	175-183	**Y**LKDGGFAF	Domain I	F/B	Y^na^KD
**E6**	A01	194-202	TL**DE^+^**M**RHF**Y	Domain I	B	D, E, H, F
**E7**	A01	198-207	M**RHF**YK**D**N**KY**	Domain I	F/B	H, F, D, K, Y
**E8**	B44	327-335	N**E**F**P**A**I**DLF	Domain II	F/B	E, P, I
**E9**	A02	339-346	KLVFELS*A	Domain II	F	E, L
E10	A01	389-397	RYKS**H**G**K**GY	Domain II	F	H, K, Y
E11	A02	405-414	ET**Q**KCEIFNV	Domain II	B	E, T, Q, N
**E12**	A02	406-414	T**Q**KCEIFNV	Domain II	F	T, Q, N
E13	A02	410-419	EIFNVK*PTCL	Domain II	F	NK
**E14**	B44	520-528	NEVVVKE*EY	Domain III	F	VEE

Variable amino acids are highlighted in bold.

*Indicated that there is only one variant of <0.001% frequency

**E^+ ^**is essential for binding of growth inhibitory MAb 1F9

F = front, B = back, F/B = wraps from front to back

Y^na ^Accessibility not available for amino acid Y

Confirmed epitope numbers are indicated in bold

**Figure 5:**
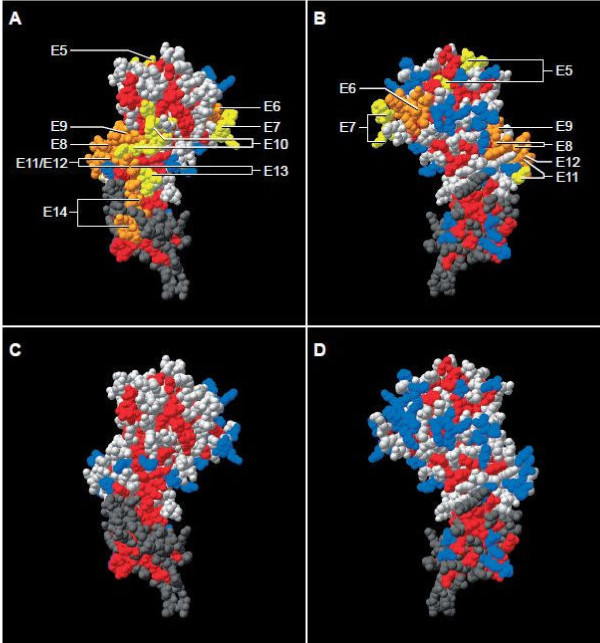
**Location within model of AMA1 crystal structure of 10 predicted HLA-A and HLA-B epitopes**. Spatial distribution of CD8 epitopes on the 3 D model structure of AMA1. The structure of PfAMA1 Domain I and II (1Z40) was fitted on the structure of PvAMA1 Domain I, II and III (1wk8), using Swiss Pdb-Viewer software http://www.expasy.org/spdbv/. Domain I and II of the PfAMA1 structure (light grey regions) and Domain III of PvAMA (dark grey region) are visible. Panels A & C show front view (largely conserved), B & D show back view (more polymorphic). Confirmed CD8 epitopes (E6, E8, E9, E12, E14) are orange in panels A & B, non-confirmed epitopes (E5, E7, E10, E11, E13) are yellow, while overlapping confirmed epitopes and non-confirmed epitopes are also in orange. Residues that are conserved among malaria species are shown in red and those that are polymorphic are shown in blue. Light grey (PfAMA) and dark grey (PvAMA1) are residues that differ between these species. Arrows point to individual epitopes. Overlapping epitopes may be indicated by multiple arrows. Confirmed epitopes E1 to E4 (see text) are not shown, as the part of the protein where these epitopes reside is not present in this crystal structure model.

Based on the sequence of AMA1[26], epitopes E2, E3, E4, E5, E6, E7, E8, E10, E11 and E12 are variable, E9, E13, E14 have a low frequency (< 0.001%) of variable residues, whereas E1 is conserved. There are disulphide bonds between several cysteines (C) that form loops; epitopes E5, E6 and E7 are contained in a C-C loop in Domain I, and E8, E9, E10, E11, E12 and E13 are in a C-C loop in Domain II. Since all of these epitopes in C-C loops are variable, it is likely that they are exposed on the surface[32]. This was confirmed by mapping these epitopes to a model structure of AMA1, constructed from *P. falciparum *Domains I and II, and *P. vivax *Domain III[28,31,32]. The signal and prodomain containing E1-E4 were not contained in this model. The localization of epitopes E5-E14 is shown in Figure 5 and Table 7. Epitopes E8, E9, E10, E12, E13 and part of E14 form a continuous cluster on the front face, E5, E7, E8 and E9 wrap around from the front to back faces, and E6 (largely) and E11 localize to the back face. E5, though not confirmed as an epitope, is largely non-variant and immediately adjacent to the conserved hydrophobic cleft, whereas E6 is in a hypervariable region of Domain I also adjacent to the cleft. E7 is contiguous with E6 as might be expected from their overlapping sequences (Table 7). E9 is close to the binding site of inhibitory MAb 4G2 and contiguous with E10 that contains R_389 _that is essential for 4G2 binding[20]. E12 appears to be clustered with E8 and E9 although it is 60 amino acids C-terminal to E9, and close to E13. As expected from the linear sequences E11 appears to be adjacent to E12 but on the back face. Unexpectedly from the linear sequence, E14 is close to E13 and adjacent to the large cluster formed by E8, E9, E10, E12, E13 and part of E14.

### Epitope polymorphism and surface accessibility

The polymorphic residues in all 14 epitopes are shown in Table 7. Only E1 is completely non-variant, although the frequencies of single amino acid polymorphisms in E9, E13 and E14 are rare (<0.001%). Overall there are 27 polymorphic residues in all 14 epitopes. For E5-E14 that could be mapped, there are 18 polymorphic residues of which 16 are surface accessible (Table 7). Thus, E6 contains variable amino acids at positions D_196_, E_197_, R_199_, H_200 _and F_201_, and all except R_199 _are accessible to the surface. Overall, R_199 _and S_345 _in these epitopes are polymorphic but not surface accessible. However, there are 10 amino acids that are surface accessible that are not polymorphic such as T_406 _and N_413 _in E12. Therefore polymorphic residues are mostly surface accessible, but there are other surface accessible residues that are not polymorphic.

## Discussion

The aim of this study was to identify class I epitopes underlying the CD8+ T cell IFN-γ responses observed on ELISpot and ICS assays following administration of the NMRC-M3V-Ad-PfCA adenovirus-vectored *P. falciparum *malaria vaccine. Mapping class I epitopes was pursued to better understand the immune responses induced by AMA1 and to guide the development of a broadly protective malaria vaccine for a genetically diverse human population.

To map the epitopes, low to mid level HLA typing was performed to determine the HLA A and B supertype for each of five immunized volunteers. Subsequently, all possible 8-10 amino acid sequences from 15-mer AMA1 peptides inducing recall responses on ELISpot assay were evaluated *in silico *using NetMHC software, and ranked for binding affinity to the A and B alleles. Predicted top-binding epitopes were synthesized and ELISpot and ICS assays were used to confirm epitope identification by demonstrating that (1) the predicted epitope recalled IFN-γ responses as effectively as the parent peptide pool, (2) the responses of the parent 15-mer peptide were CD8+ T cell-dependent by ELISpot assay, and (3) the epitopes themselves stimulated IFN-γ production by CD8+ T cells on ICS assay.

Using this approach, sixteen 15-mer peptides were down-selected from the peptide pools on the basis of stimulating positive recall responses by ELISpot assay from the immunized volunteers. NetMHC predicted 14 class I 8-10-mer epitopes lying within these peptides for the five volunteers. Nine of these were shown by ELISpot assay to be active (E1, E2, E3, E4, E6, E8, E9, E12 and E14), eliciting IFN-γ responses comparable to those elicited by the parent peptide pools, while four could not be adequately assessed as the PBMC eliciting strong recall responses for these volunteers were no longer available (E5, E7, E11, E13), and one induced low recall responses relative to the parent peptide pool (E10).

To further evaluate the predicted epitopes, ELISpot depletion assays were conducted by stimulating PBMC from four volunteers with three parent peptide pools (Ap1, Ap4, Ap10) or with five individual 15-mer peptides from those pools (1, 3, 5, 7, 16). The 15-mers contained, respectively, the epitopes E1, E3, E4, E6 and E14. CD8+ T cell depletion reduced responses in five tests of the three peptide pools by 65%, 97%, 96%, 100% and 100%, respectively, and to the five 15-mers by 56-100% (mean of 88%). In contrast, depleting CD4+ T cells increased activity in Ap1 and two of the three peptides it contained as well as one peptide in Ap10, and reduced the ELISpot activity recalled by the remaining peptides by only 18- 37% (mean of 30%), strongly supporting the contention that the selected 15-mers contained minimal class I epitopes.

ICS assays performed using six minimal epitopes showed that four could recall IFN-γ responses from CD8+ T cells (E3, E6, E8 and E14), while two could not (E10 and E11). In contrast IFN-γ responses from CD4 cells were minimal or absent when stimulating with the same minimal epitopes.

In summary, of the nine epitopes active by ELISpot (E1, E2, E3, E4, E6, E8, E9, E12 and E14), subsequent ELISpot depletion assays support the identification of E1, E3, E4, E6 and E14 and ICS assays the identification of E3, E6, E8 and E14, while E2, E9 and E12 could not be adequately assessed due to PBMC supplies. Four additional epitopes (E5, E7, E11 and E13) have yet to be confirmed as class I-restricted by testing for reductions in ELISpot responses on CD8+ T cell depletion or by testing for CD8+ T cell responses on ICS assay. The remaining epitope, E10, recalled only 10% of the response recalled by the parent peptide pool on ELISpot assay and was not active on ICS assay, and thus may not be correctly identified.

While the focus of these experiments was induction of IFN-γ in ELISpot or ICS assays, four epitopes (E3, E6, E8 and E14) demonstrated recall of multifunctional responses (production of at least two cytokines of IFN-γ, TNF-α and IL-2) that might, due to the higher IFN-γ secretion levels in multifunctional T cells, contribute disproportionately to the overall IFN-γ response at 1 month following immunization, acting as more potent immune effectors.

### Developing a reagent for class I responses to AMA1

The 14 putative minimal class I epitopes were combined into a reagent pool, denoted AMA1-14e, and this pool was shown to be an efficient stimulant for recalling T cell responses to AMA1. The AMA1-14e pool stimulated responses equivalent to parent 15-mer peptides and peptide pools, and these responses were CD8+ T cell-dependent by ELISpot depletion assay. Future trials with the NMRC-M3V-Ad-PfCA vaccine should reveal the usefulness of the AMA1-14e reagent. A previous study using DNA plasmids containing five malaria candidate vaccine genes relied on using HLA-matched peptides[62]; an HLA-diverse reagent similar to AMA1-14e would have greatly simplified this approach.

### Population coverage associated with the identified epitopes

The 14 epitopes identified in this study were characterized with regard to the coverage provided for the HLA diverse human population. The epitopes were restricted by four of the seven HLA supertypes expressed by the volunteers: A01, A02, B08 and B44. MHC class 1 molecules from the other supertypes identified in the volunteers, A03, B27 and B58, may also have recognized AMA1 CD8+ T cell epitopes. Such epitopes binding to these allelic supertypes were predicted by NetMHC, but were not located within the 15-mers recalling the strongest responses by ELISpot assay, and thus were not synthesized or tested. A01, A02, B08 and B44 supertypes cover approximately 100% of Caucasian populations and 27% of US African Americans [61]. Therefore, to reach nearly 100% coverage, particularly of sub-Saharan Africans, other epitopes restricted by the prevalent supertypes A03, A24, B07 and B58 need to be identified. Previous observations identified 11 proliferative T cell epitopes in Kenya[36], one of which was associated with lower risk of parasitaemia[43]. Since these were long peptides (12-22 amino acids) they may contain CD4+ or CD8+ T cell epitopes.

### Epitope variability and location within the AMA1 crystal structure

The structures of Domains I, II and III of *P. falciparum *AMA1 are relatively conserved among *Plasmodium *species [26,28], but there is nevertheless extensive polymorphism present as well [63-65], reflected by the strain-specificity of inhibitory antibodies [22,26,66]. Many of the previously defined proliferative epitopes (Table 2) map to variant regions of the protein [36,43]. The 14 putative class I epitopes predicted in this study were compared to available sequence data on AMA1 [26], and this analysis showed that 13 of the 14 epitopes likewise contained one or more polymorphic amino acids (all except E1), although three were variable at a low level (<0.001%) (E9, E13 and E14).

A model of the crystal structure of AMA1 was generated to allow localization of ten of the 14 epitopes within the tertiary structure (Figure 5). This is one of the first studies in which CD8+ T cell epitopes, rather than antibody epitopes, have been localized in this way. Many of the ten epitopes clustered together on the front face, whereas a few wrapped around from the front to back or were localized on the back face. Surface location was a surprising finding, since it is generally thought that class I-restricted epitopes occur independently of surface exposure. Surface location of T epitopes has been previously described in HIV where it was suggested that this resulted in increase accessibility to the antigen-processing pathway [67].

Epitope E6 localized to a hyper-variable region of AMA1 on the back of the molecule, adjacent to the putative hydrophobic cleft[32] and E_197_, one of the variable residues in E6 (see E_197 _in Table 7), is crucial to the binding of the growth inhibitory MAb 1F9 and also forms part of a putative AMA1 receptor[35]. E_197 _is also contained within the variable epitope 189 identified in Kenya[36].

Amino acid polymorphism in AMA1 is associated with surface accessibility, whether on the front or back face. Immune pressure may have driven polymorphism in residues surrounding the putative binding site in the hydrophobic cleft[31]. Other studies have attributed polymorphism within T cell epitope regions to host evasion of T cell recognition irrespective of molecular function [26,34,68] in which parasite strains are favored through reduced affinity of molecular sequences for binding to HLA A or B alleles[31]. An association between immune pressure and T cell epitope variability has been proposed for other malaria antigens such as CSP and will need to be overcome for successful vaccine development[14,34]. In one study, a diversity-covering approach that took into account genetic linkages among polymorphic sequences improved allelic recognition by ELISA and growth inhibitory antibodies for AMA1[69], and may also be applicable to T cell-dependent AMA1 vaccines.

Four HLA-B8/B44-restricted epitopes are close to the N-terminal in the signal sequence and prodomain (E1-E4), while seven of the 10 remaining epitopes are HLA-A1/A2-restricted. The signal sequence and prodomain are cleaved off first during merozoite release and red cell invasion[70] during which AMA1 re-localizes around the merozoite surface, and then Domains I and II and part of Domain III are shed as the merozoite invades[26]. It is therefore possible that this sequence of events influences AMA1 uptake by antigen-presenting cells, such that epitopes in the signal sequence and prodomain may be presented to HLA-B-restricted T cells, while the later shedding of the domains favors presentation to HLA-A-restricted T cells. It is also interesting that the single non-polymorphic epitope E14 is located in the part of Domain III which remains on the merozoite during invasion.

## Conclusions

This study identified fourteen putative minimal CD8+ T cell-dependent epitopes within AMA1 that are restricted by four HLA supertypes that together are expressed by 100% of Caucasians, but only 27% of African Americans. Nine epitopes were confirmed using ELISpot or ELISpot and ICS assays. Some of these clustered on the front face of AMA1, while others were more randomly distributed but still mostly surface accessible. When the 14 epitopes were mixed together, they recalled CD8+ T cell responses in volunteers with different HLA supertypes providing a reagent useful for measuring CD8+ T cell responses in genetically different populations.

Further testing of the NMRC-M3V-Ad-PfCA vaccine in volunteers representing different HLA A and B supertypes would provide the opportunity to identify additional class I epitopes. This could support the design of a chimeric AMA1 molecule able to elicit broadly protective responses among an HLA diverse population. Determining the potential contribution of these epitopes for inducing protective responses awaits future testing of the NMRC-M3V-Ad-PfCA vaccine in challenge studies.

## Competing interests

JTB, CRK and DLD are inventors listed on U.S. Patent No., U.S. Patent No. 2009-0148477 A1, and international patent application PCT/US06/33982, titled "Adenoviral Vector-based Malaria Vaccines"; JTB, CRK, TLR and DLD are inventors listed on U.S. Patent Application 12/522,335, and international patent application PCT/US08/50565 titled "Adenoviral Vector-based Malaria Vaccines". JTB, GenVec, Inc. contributed to the design of the study, interpretation of data, editing of drafts and final manuscript approval but he did not contribute to protocol development or study execution.

## Authors' contributions

MS and TLR designed the research; TLR, DLD, JB and CRK designed the vaccine; HG, JL, EA, GB, MB, and RS performed ELISpot assays; SM and FF performed the ICS assays; YK, BP and AS used NetMHC to predict epitopes; CO, CT, DR, and IC were investigators in the clinical trial; BF and ER performed the crystal localization studies; MS, YK, DLD, TLR and MRH wrote the paper. All authors read and approved the final manuscript.
